# ﻿Four new species of *Beltraniella* (Amphisphaeriales, Beltraniaceae) revealed by morphology and phylogenetic analyses from China

**DOI:** 10.3897/mycokeys.116.140506

**Published:** 2025-04-09

**Authors:** Wen-Wen Liu, Chang-Zhun Yin, Zhao-Xue Zhang, Xing-Sheng Wang, Zhe Meng, Xiu-Guo Zhang, Shi Wang

**Affiliations:** 1 College of Life Sciences, Shandong Normal University, Jinan, 250358, China Shandong Normal University Jinan China; 2 Shandong Provincial Key Laboratory for Biology of Vegetable Diseases and Insect Pests, College of Plant Protection, Shandong Agricultural University, Taian, 271018, China Shandong Agricultural University Taian China

**Keywords:** Dematiaceous hyphomycetes, novel taxa, phylogeny, Sordariomycetes, taxonomy

## Abstract

*Beltraniella* is a widely-distributed genus on Earth, although its abundance is relatively limited in relation to other dematiaceous hyphomycetes. In the present study, diseased leaves of *Myristicafragrans* and decaying leaves were collected from Hainan and Sichuan Province. Fungal DNA was amplified and sequenced using two barcodes, the internal transcribed spacer (ITS) and large subunit of ribosomal RNA (LSU), and phylogenetic analyses were conducted through maximum likelihood (ML) and Bayesian inference (BI) algorithms. Four new species of *Beltraniella*, *B.dujiangyanensis*, *B.jianfengensis*, *B.myristicae*, and *B.xinglongensis* are identified through phylogenetic analyses and morphological comparison during a survey of fungal diversity in Hainan and Sichuan Provinces, China. Detailed descriptions of the morphological characteristics of these four new species are provided and illustrated with figures.

## ﻿Introduction

*Beltraniella* was proposed by Subramanian in 1952, and he selected *B.odinae* as the type species (Subramanian 1952). Currently, a total of 33 epithet records of *Beltraniella* have been documented in the Index Fungorum (http://www.indexfungorum.org/, accessed on 14 February 2025). *Beltraniella* belongs to the Sordariomycetes, Amphisphaeriales, Beltraniaceae (Hyde et al. 2024). *Beltraniella* was characterized by sterile setae, which were extensions of conidiophores, or were present among conidiophores and arising from radially lobed basal cells. Conidiophores were branched, often with setae-shaped apices, and they originated from radially lobulated basal cells. Conidiogenous cells were polyblastic and sympodial; Conidia were turbinate or biconical. The distinction between setae and conidiophores lies in their apex; setae gradually narrow to a sharp point, whereas conidiophores may sometimes be reduced to conidiogenous cells (Hyde et al. 2020b). Furthermore, *Beltraniella* is a genus of dematiaceous hyphomycetes that play a crucial ecological role in natural ecosystems by breaking down lignin and cellulose, recycling matter and energy, and maintaining ecosystem balance.

*Beltraniella* typically inhabits decaying leaves and other natural substrates on the ground, maintaining the balance of natural ecosystems, and aiding in the decomposition of diseased or decaying leaves (Dighton et al. 1985). Shirouzu et al. (2010) isolated and identified *B.botryospora* Shirouzu & Tokum, a fungus frequently reported on both live and deciduous leaves of *Quercusaspera*, suggesting a close relationship with this species. Hyde et al. (2020a) reported a new species, *B.ramosiphora* C.G. Lin & K.D. Hyde, found in decomposing organic matter on decaying leaves, while Tan and Roger (2022) reported *B.hesseae* Y.P. Tan, Bishop-Hurley & R.G on the leaves of *Digitariaciliaris*. The principal methodology employed in studying this fungus encompasses a synthesis of traditional morphological taxonomy and molecular systematics. Crous et al. (2014) isolated and characterized *B.endiandrae* Crous & Summerell, reporting that its conidia were solitary, light brown, and smooth, with hyaline transverse bands. They also demonstrated the colony color variation of *B.endiandrae* on three distinct media. The frontal and reverse sides appeared iron-gray on PDA medium; the light olive-gray patches appeared on OA medium, while the frontal side was light olive-gray and the reverse side was yellowish-brown on MEA medium. Tibpromma et al. (2018) reported two new species, *B.pandanicola* Tibpromma & K.D. Hyde and *B.thailandica* Tibpromma & K.D. Hyde [as ‘*thailandicus*’], and described their morphological characteristics in detail. In addition, they provided a detailed description of *Pandanus* growing on withered leaves, whereas Crous et al. (2016) employed the ITS sequence to conduct a phylogenetic analysis of *Beltraniella*. Recently, Liu et al. (2024) identified a new species named *B.jiangxiensis* P. Razaghi, Raza & L. Cai, through phylogenetic analysis based on ITS and LSU sequences, combined with morphological analysis. This method is currently widely accepted as the identification method for *Beltraniella*.

The primary objective of this study is to identify putative new strains of *Beltraniella* through morphological comparison and phylogenetic analysis. Four new species of *Beltraniella* were identified and thoroughly characterized, with their differences from closely related species compared and discussed, thereby enriching the species diversity of the genus.

## ﻿Materials and methods

### ﻿Sample collection and treatment

Samples of diseased or decaying leaves were collected in Hainan and Sichuan provinces from June 2023 to March 2024. Upon collection, they were numbered by time, location and plant type, and then photographed and recorded. Flatter leaves were chosen for photography. The processed samples were returned to the kraft bag for the next step. For each sample, 4–7 diseased or decaying leaves were cut into squares (5 × 5 mm) and placed in sterile containers. These were then sterilized on a clean bench. Pour in 75% alcohol, soak the leaves thoroughly for 1 minute to sterilize their surfaces. Use a disposable syringe to remove the alcohol, rinse with sterile water, then remove the sterile water and add 5% sodium hypochlorite to sterilize the leaf surface again for 30 seconds. Rinse the leaves three times with sterile water, then place them on sterilized filter paper to dry using sterilized tweezers. Once dry, clip the leaves with the diseased spot pointing downward and place 3–5 samples of them on each PDA medium (PDA: 14 g agar, 20 g dextrose, 200 g potato, 1000 mL distilled water, pH 7.0). The medium with leaves was securely wrapped with sealing film and placed in a constant temperature incubator at 25 °C for incubation. The growth of the fungus was observed every day, and after 2–3 days of incubation, the agar with fungal growth was transferred from the PDA medium to a new PDA medium for purification.

### ﻿Morphological and cultural characterization

The single colonies that were isolated and purified were photographed on the 7^th^ and 14^th^ day of growth, using a digital camera (Canon Powershot G7X; Beijing, China), on both the surface and reverse of the PDA medium. A stereo microscope (Olympus SZX10; Beijing, China) was used to observe whether conidia were produced. If conidia were observed, a temporary mount was prepared to examine the morphology of the fungal conidia under a microscope (Olympus BX53). Subsequently, fungal structures, including conidia and conidiogenous cells, were photographed using a high-definition digital camera (Olympus DP80). All strains were stored in a 4 °C thermostat using sterilized 10% glycerol test tubes. Voucher specimens have been carefully preserved in two herbariums: the Herbarium of the Department of Plant Pathology at Shandong Agricultural University in Taian, China (HSAUP), and the Herbarium Mycologicum Academiae Sinicae at the Institute of Microbiology, Chinese Academy of Sciences in Beijing, China (HMAS). Additionally, living cultures derived from the holotype have been safeguarded in the Shandong Agricultural University Culture Collection (SAUCC). The morphological description and taxonomic characters of the new species have been uploaded to MycoBank (http://www.mycobank.org).

### ﻿DNA extraction, PCR amplification, and sequencing

The method of extracting fungal DNA involves using CTAB (cetyl trimethyl ammonium bromide) (Wang et al. 2023). When the mycelium has grown to a certain degree in the PDA medium, use a sterilized scalpel to scrape approximately 0.2 g of mycelium into a 1.5 mL centrifugal tube, add precipitating CTAB lysate to the tube, pulverize the mycelium using a grinder, and then place the tube in a water bath at 65 °C for 2 hours. After pulverization, centrifuge the sample to extract the supernatant, add chloroform (and other precipitate agents) to the supernatant to isolate the genomic DNA. Further centrifuge the supernatant, and then add chloroform: isoamyl alcohol (24:1) to precipitate the DNA (Doyle and Doyle 1990; Guo et al. 2000). PCR (Polymerase Chain Reaction) amplification of the extracted fungal DNA is performed using ITS and LSU (White et al. 1990; Glass and Donaldson 1995). Each sterilized PCR tube contains a total of 25 μL reaction mixture, which includes 9.5 μL of ddH_2_O, 12.5 μL of 2 × Taq Plus Master Mix (Shanghai, China) (with dye) (Yeasn Biotechnology, Shanghai, China, Cat No. 10154ES03), 1 μL of forward primer, and 1 μL of reverse primer. The products of PCR amplification were detected by electrophoresis in a 2% agarose gel. After electrophoresis, the gel was removed and observed under UV light, where the presence of DNA was indicated by fluorescent bands (Zhang et al. 2022). PCR primer synthesis and DNA sequencing were completed by Tsingke Biotechnology Co., Ltd. (Qingdao, China). Once sequencing was completed, MAGE7 (Kumar et al. 2016) was utilized for sequence comparison and splicing of the sequencing results. The gene sequences of the four new species were uploaded to the GenBank. Subsequently, the most recent article was downloaded by searching for ‘*Beltraniella*’ on Index Fungorum (https://indexfungorum.org/Names/Names.asp, accessed on 14 February 2025). The GenBank table mentioned in the article was found, and the results were presented in Table 1.

**Table 1.** GenBank numbers used in the phylogenetic analysis of *Beltraniella*.

Species	Strains	Country	GenBank accession numbers	
			ITS	LSU
*Beltraniaquerna*	CBS 126097	Spain	MH864016	MH875474
*Beltraniapseudorhombica*	CBS 138003*	China	MH554124	NG_058667
*Beltraniellaacaciae*	CPC 29498*	USA	NR_147685	KY173483
*Beltraniellabotryospora*	TMQa1A18	Japan	N/A	AB496426
*Beltraniellabrevis*	DS 2-23	China	MN252876	MN252883
*Beltraniellacarolinensis*	9502 (IFO)	N/A	N/A	DQ810233
***Beltranielladujiangyanensis***	SAUCC427003*	**China**	**PP301351**	**PP301362**
***Beltranielladujiangyanensis***	**SAUCC427004**	**China**	**PP301352**	**PP301363**
*Beltraniellaendiandrae*	CBS 137976*	Australia	NR_148073	KJ869185
*Beltraniellaendiandrae*	CBS 137976	Australia	KJ869128	MH878615
*Beltraniellafertilis*	MFLUCC 20-0119	Thailand	MT835158	MT835156
*Beltraniellafertilis*	MRC 3BEL	Thailand	MF580247	MF580254
*Beltraniellahesseae*	BRIP 72433a*	Australia	OP023124	OP023141
*Beltraniellahumicola*	CBS 203.64	India	MH858416	MH870044
***Beltraniellajianfengensis***	SAUCC639001*	**China**	**PP301353**	**PP301364**
***Beltraniellajianfengensis***	**SAUCC639002**	**China**	**PP301354**	**PP301365**
*Beltraniellajiangxiensis*	CGMCC 3.23486*	N/A	OP022178	OP022174
***Beltraniellamyristicae***	SAUCC638601*	**China**	**PP301355**	**PP301366**
***Beltraniellamyristicae***	**SAUCC638602**	**China**	**PP301356**	**PP301367**
*Beltraniellapandanicola*	MFLUCC 18-0121*	Thailand	MH275049	MH260281
*Beltraniellapodocarpi*	CPC 36783*	South Africa	MT373370	NG_074446
*Beltraniellaportoricensis*	CBS 856.70	N/A	MH859981	MH871777
*Beltraniellapseudoportoricensis*	CBS 145547*	South Africa	NR_165552	NG_067875
*Beltraniellaramosiphora*	MFLU 17-2649*	Thailand	NR_171732	NG_073615
*Beltraniellathailandica*	MFLUCC 16-0377*	Thailand	NR_168175	NG_068824
*Beltraniellaxinglongensis*	SAUCC737701*	China	PQ325612	PQ325618
*Beltraniellaxinglongensis*	SAUCC737702	China	PQ325613	PQ325619

Notes: New species established in this study are shown in bold. Those marked “*” in the table are represented as ex-type or ex-epitype strains. N/A: Not available.

### ﻿Phylogenetic analyses

Nucleic acid sequences of *Beltraniella* were downloaded from the National Center for Biotechnology Information (https://www.ncbi.nlm.nih.gov/, accessed on 14 February 2025), and GenBank accession numbers were obtained from the latest version of the article (Zhang et al. 2000). Nucleic acid sequences of the four new species were aligned with reference sequences from the literature using MAFFT 7 (http://mafft.cbrc.jp/alignment/server/, accessed on 14 February 2025) (Katoh et al. 2019). Data from the completed sequence alignments were systematically analyzed using the maximum likelihood (ML) and Bayesian inference (BI) methods. BI and ML analyses were conducted separately through registering on the CIPRES website (Miller et al. 2012). For the ML analysis, RAxML-HPC2 v.8.2.12 was used on XSEDE with 1000 rapid bootstrap replications and the GTRGAMMA model (Stamatakis 2014). MrModeltest v.2.3 (Nylander 2004) software was utilized to screen for optimal evolutionary models, while BI was conducted using MrBayes 3.2.7a (on XSEDE) (Huelsenbeck and Ronquist 2001; Ronquist and Huelsenbeck 2003; Ronquist et al. 2012). FigTree v1.4.3 (http://tree.bio.ed.ac.uk/software/figtree/, accessed on 14 February 2025) was used to open the successfully obtained topology and reroot the tree with the outgroup. The final phylogenetic tree was created with Adobe Illustrator CC 2019. In the final phylogenetic tree output, the names and strain numbers of the new species are marked in red.

## ﻿Results

### ﻿Phylogenetic analyses

**Figure 1.**
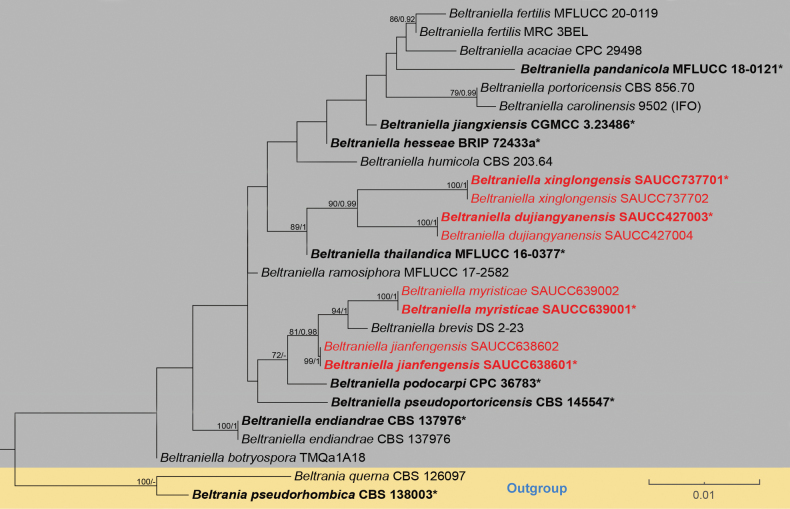
Phylogenetic tree of *Beltraniella* based on combined ITS and LSU sequences. Bootstrap support values exceeding 70% (ML) and 0.90 (BI) are indicated by MLBS/BPP, and new species are highlighted in red. Branches separated by gray and yellow indicate different species of *Beltraniella* and *Beltrania*. The lines in the lower right-hand corner represent changes of 0.01 nucleotides per site.

Interspecific relationships of the genus *Beltraniella* were identified by phylogenetic analyses. These analyses were based on downloaded sequences and newly acquired sequences of new species, using *Beltraniapseudorhombica* Crous & Y. Zhang ter CBS 138003 and *B.querna* Harkn CBS 126097 as outgroups. The concatenated sequence matrix comprised 27 sequences with a total of 1295 characters (the combined dataset: ITS: 1–502, LSU: 503–1295). There were 1192 constant characters, 33 variable but parsimony non-informative, and 70 parsimony informative characters. The topologies of the evolutionary trees obtained using the maximum likelihood (ML) and Bayesian inference (BI) algorithms are essentially similar. Fig. 1 shows the best-scoring maximum likelihood (ML) evolutionary tree, where maximum likelihood bootstrap analyses and Bayesian posterior probabilities (MLBS/BPP) are labeled at node positions. Eight new strains of *Beltraniella* were incorporated into the phylogenetic analysis presented in the ML tree. The eight new strains introduced in this study were divided into four monophyletic branches in the phylogenetic tree, representing four new species of *Beltraniella*, *B.dujiangyanensis*, *B.jianfengensis*, *B.myristicae*, and *B.xinglongensis*. The strains of *Beltranielladujiangyanensis* form a distinct clade sister to *B.xinglongensis* with good bootstrap support (ML/BI = 90/0.99); *B.thailandica* forms a high-support clade (ML/BI = 89/1.00) alongside the lineage consisting of *B.dujiangyanensis* and *B.xinglongensis*; *B.myristicae* forms a high-support clade (ML/BI = 94/1.00) with *B.brevis*; and *B.jianfengensis* forms a high-support clade (ML/BI = 81/0.98) with the lineage consisting of *B.brevis* and *B.myristicae*.

### ﻿Taxonomy

#### ﻿ Beltraniella dujiangyanensis

Taxon classificationFungiAmphisphaerialesBeltraniaceae

W.W. Liu, C.Z. Yin, Z.X. Zhang & X.G. Zhang sp. nov.

51C52E17-FCFA-582D-AA9B-72D391338673

MB853427

Fig. 2

##### Holotype.

China • Sichuan Province, Dujiangyan City, 30°57'53"N, 103°35'13"E, on decaying leaves, 24 June 2023, W.W. Liu, holotype HMAS 352921, ex-type living culture SAUCC427003.

**Figure 2.**
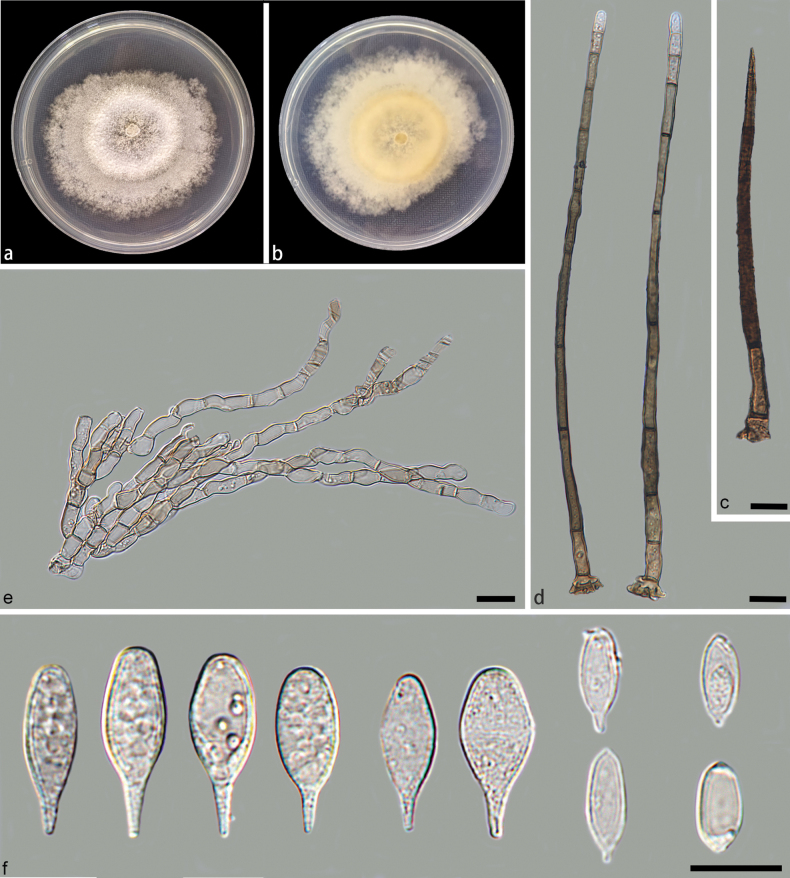
*Beltranielladujiangyanensis* (holotype: HMAS 352921) **a, b** colony front and back after 7 days culture on PDA **c** setae **d** long conidiophores **e** short conidiophores **f** separating cells and conidia. Scale bars: 10 μm (**c–f**).

##### Etymology.

The epithet “*dujiangyanensis*” denotes the geographical origin of the strains, namely, Dujiangyan City.

##### Description.

Parasitic on decaying leaves. Asexual morph: Setae unbranched, straight or flexuous, single, dark brown, subulate, thick-walled, tapering to a pointed apex, 9–10septate, verrucose, dark brown, swollen, arising from a radially lobed basal cell, 83.9–150.2 × 3.0–5.4 µm. Conidiophores hyaline, presenting two distinct forms: long and short. Long conidiophores arise from lobed basal cells, macronematous, erect, straight or slightly curved, either simple or rarely branched, septate, verrucose, dark-brown, apical part lighter, arising from basal cells of setae or from separate cells, 113.1–259.9 × 3.1–5.8 μm. Short conidiophores hyaline, septate, smooth edges, simple or branched, 13.1–31.9 × 3.2–5.7 µm. Conidiogenous cells polyblastic, integrated, determinate, cylindrical, smooth, terminal, geniculate, denticulate, hyaline to subhyaline, 5.5–10.9 × 2.9–4.7 µm. Separating cells ellipsoid to subglobose, smooth, subhyaline, single, denticle at each end, 9.7–12.3 × 3.1–5.3 µm. Conidia originate directly from the conidiogenous cells in the long conidiophores and from the separating cells in the short ones. Conidia arise directly from conidiogenous cells or from separating cells, simple, teardrop-shaped, sometimes verrucose, narrow-tipped, terminal, hyaline, smooth, straight, rostrate to pointed at proximal end, truncate at distal end, 16.5–21.1 × 4.2–8.5 µm. Sexual morph: Inconclusive.

##### Culture characteristics.

On PDA medium, after seven days of dark incubation in a 25 °C incubator, colonies reached 68 mm in diameter with a growth rate of 9.2–10.2 mm/day. Colonies on PDA medium were concentric, flatter, white, moderately dense, granular surface, sparse aerial mycelia, with mycelium in the middle portion aggregated into a circle and mycelium on the edges dispersed to form a fluffy shape; reverse, pale yellow to white, fluffy edges.

##### Additional material studied.

China • Sichuan Province, Dujiangyan City, 30°57'53"N, 103°35'13"E, on decaying leaves, 24 June 2023, W.W. Liu, HSAUP 427004, living culture SAUCC427004.

##### Notes.

Based on the phylogenetic tree constructed using ITS and LSU sequence data, *Beltranielladujiangyanensis* was identified as the closest relative to *B.xinglongensis* sp. nov., with 90% MLBS and 0.99 BPP support values (Fig. 1). Additionally, there is a disparity of 16/502 bp between their ITS sequences. Morphologically, *B.dujiangyanensis* differed from *B.xinglongensis* in having shorter long conidiophores (*B.dujiangyanensis*: 113.1–259.9 × 3.1–5.8 μm vs. *B.xinglongensis*: 232.5–298.6 × 2.4–4.9 μm) and fewer septa (*B.dujiangyanensis*: 9–10 septa vs. *B.xinglongensis*: 13–15 septa), shorter in short conidiophores (*B.dujiangyanensis*: 13.1–31.9 × 3.2–5.7 μm vs. *B.xinglongensis*: 21.2–47.8 × 3.2–6.4 μm), shorter separating cells (*B.dujiangyanensis*: 9.7–12.3 × 3.1–5.3 μm vs. *B.xinglongensis*: 13.6–17.6 × 2.3–5.4 μm) and shorter conidia (*B.dujiangyanensis*: 16.5–21.1 × 4.2–8.5 μm vs. *B.xinglongensis*: 21.9–28.7 × 5.0–9.5 μm). As a result, *B.dujiangyanensis* was identified as a new species of *Beltraniella* by phylogenetic analysis and morphological comparison.

#### ﻿ Beltraniella jianfengensis

Taxon classificationFungiAmphisphaerialesBeltraniaceae

W.W. Liu, C.Z. Yin, Z.X. Zhang & X.G. Zhang sp. nov.

BAF93889-A69B-527A-8A54-6E9AF089C4EC

MB853429

Fig. 3

##### Holotype.

China • Hainan Province, Ledong County, Jianfengling National Forest Park, 18°44'25"N, 108°51'32"E, on decaying leaves, 14 October 2023, W.W. Liu, holotype HMAS 352923, ex-type living culture SAUCC639001.

**Figure 3.**
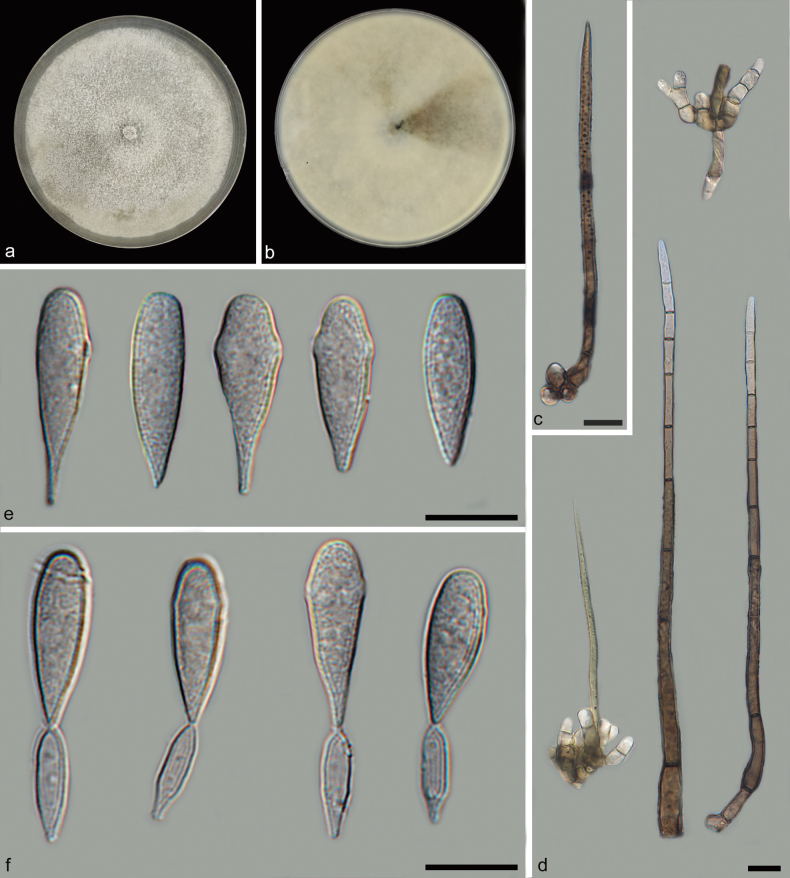
*Beltraniellajianfengensis* (holotype: HMAS 352923) **a, b** colony front and back after 7 days culture on PDA **c** setae **d** long conidiophores and short conidiophores **e** conidia **f** separating cells and conidia. Scale bars: 10 μm (**c–f**).

##### Etymology.

The epithet “*jianfengensis*” signifies the geographical location of the holotype, specifically Jianfengling National Forest Park.

##### Description.

Parasitic on decaying leaves. Asexual morph: Setae subulate, emerging from lobed basal cells, upright, straight or slightly curved, simple, septate, verrucose, dark-brown, swollen, radially lobed basal cell, 84.3–254.4 × 2.9–4.9 μm. Conidiophores macronematous, mononematous, occurring in two distinct forms: long and short. Long conidiophores arise from lobed basal cells and have a setiform appearance, upright, straight to slightly curved, simple or rarely branched, septate, verrucose, dark brown, swollen at the base, with a lighter apical region, arising from basal cells of setae or from separate ones, without a hyaline transverse band, 171.8–254.9 × 2.6–4.9 μm. Short conidiophores solitary or grouped, smoother, pale brown, smaller, 20.1–57.2 × 3.3–6.5 μm. Conidiogenous cells cylindrical, polyblastic, integrating sympodially, denticulate surface, 9.2–15.3 × 2.2–5.0 μm. Separating cells ellipsoid to subglobose, smooth, subhyaline, denticle at each end, 10.7–14.7 × 2.8–5.5 μm. Conidia originate directly from the conidiogenous cells on long conidiophores and from the separating cells on short conidiophores, turbinated, obovate to obpyriform, subhyaline, simple, smooth, straight, terminal, 17.1–23.6 × 3.6–9.5 μm. Sexual morph: Inconclusive.

##### Culture characteristics.

On PDA medium, after seven days of dark incubation in a 25 °C incubator, colonies reached 90 mm in diameter with a growth rate of 12.5–13.1 mm/day. Colonies on PDA, cottony, moderately dense, sparse aerial mycelia, steel-blue gray, granular surface, with gray exudates, flatter, smooth edge; reverse steel-blue gray, smooth edge.

##### Additional material studied.

China • Hainan Province, Ledong County, Jianfengling National Forest Park, 18°44'25"N, 108°51'32"E, on decaying leaves, 14 October 2023, W.W. Liu, HSAUP 639002, living culture SAUCC639002.

##### Notes.

Based on the phylogenetic tree of ITS and LSU sequences, *Beltraniellajianfengensis* emerged as a cluster with *B.brevis* and *B.myristicae*. However, a significant discrepancy was noted in the ITS sequence, with a disparity of 6/502 bp between *B.jianfengensis* and *B.brevis*; and a disparity of 4/496 bp between *B.jianfengensis* and *B.myristicae*. Furthermore, a substantial difference was observed in their LSU sequences. Morphologically, *B.jianfengensis* was different from *B.brevis* by having narrower setae (*B.jianfengensis*: 84.3–254.4 × 2.9–4.9 vs. *B.brevis*: 89–251 × 4.5–10.5 µm), and shorter conidia (*B.jianfengensis*: 17.1–23.6 × 3.6–9.5 vs. *B.brevis*: 20–26.5 × 4.5–7.2 µm) (Hyde et al. 2020b), and there were also differences in conidial shape: *B.brevis* exhibited diamond-shaped conidia with a hyaline supraequatorial transverse band, whereas *B.myristicae* had teardrop-shaped conidia lacking a hyaline transverse band. Additionally, *B.jianfengensis* was different from *B.myristicae* by having longer and wider separating cells (*B.jianfengensis*: 9.2–15.3 × 2.8–5.5 vs. *B.myristicae*: 8.7–12.5 × 2.5–5.4), and there were also differences in separating cells’ shape: *B.jianfengensis* features two distinct transverse projections on its surface, whereas *B.myristicae* boasts a smoother exterior devoid of such projections. Consequently, *B.jianfengensis* was classified as a new species within the genus *Beltraniella*, through a combination of phylogenetic analysis and morphological comparisons.

#### ﻿ Beltraniella myristicae

Taxon classificationFungiAmphisphaerialesBeltraniaceae

W.W. Liu, C.Z. Yin, Z.X. Zhang & X.G. Zhang sp. nov.

F0524231-4BEC-5D99-A80B-78CE76D93E3E

MB853428

Fig. 4

##### Holotype.

China • Hainan Province, Ledong County, Jianfengling National Forest Park, 18°44'25"N, 108°51'32"E, on diseased leaves of *Myristicafragrans* (Myristicaceae), 14 October 2023, W.W. Liu, holotype HMAS 352922, ex-type living culture SAUCC638601.

##### Etymology.

The epithet “*myristicae*” is derived from the name of the host plant, *Myristicafragrans*.

##### Description.

**Figure 4.**
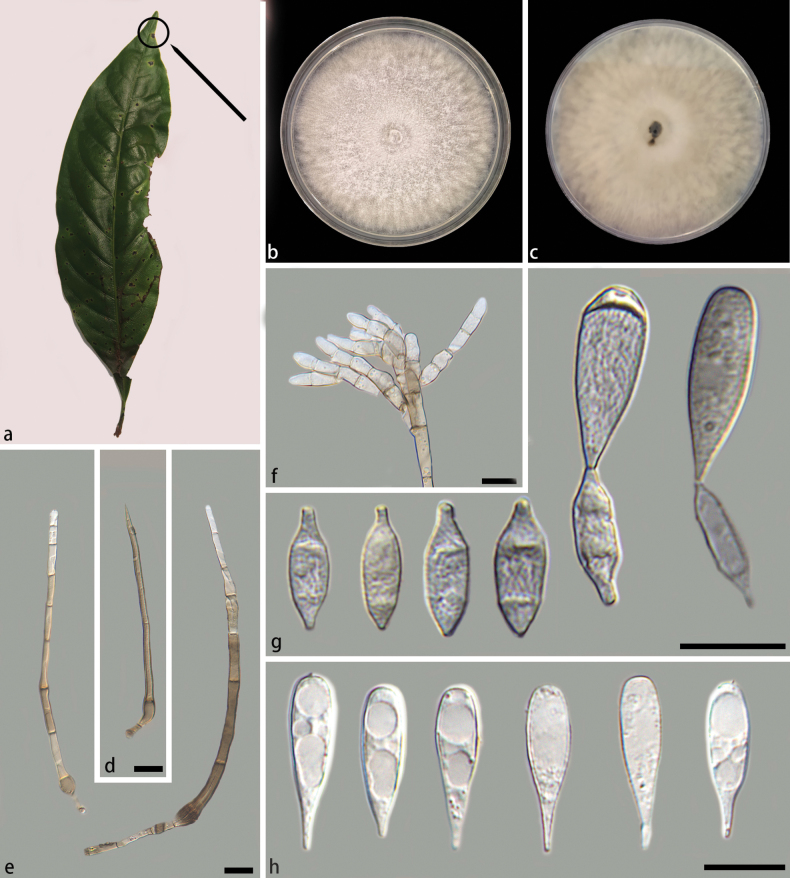
*Beltraniellamyristicae* (holotype: HMAS 352922) **a** leaf of host plant *Myristicafragrans*, black circle and arrow indicate the location of fungal infestation **b, c** colony front and back after 7 days culture on PDA **d** setae **e** long conidiophores **f** short conidiophores **g** separating cells and conidia **h** conidia. Scale bars: 10 μm (**d–h**).

Associated with diseased leaves of *Myristicafragrans*, the surface of the leaf blade shows black irregular protrusions, marked with black circles and arrows in Fig. 4. Asexual morph: Setae dark-brown, simple, subulate, verrucose, 80.5–99.8 × 2.7–5.5 µm. Conidiophores present two distinct forms: long and short. Long conidiophores emerge from lobed basal cells, macronematous, setiform, upright, straight or gently curved, simple, septate, verrucose, subhyaline to pale olivaceous, swollen at the base, arising from basal cells of setae or from separate, 74.4–150.5 × 2.9–5.3 µm. Short conidiophores hyaline, septate, smooth edges, simple or branched, 18.3–45.0 × 2.7–5.8 µm. Conidiogenous cell polyblastic, ovoid, hyaline, 5.5–12.5 × 2.0–4.9 µm. Separating cells ellipsoid to subglobose, smooth, 7.0–12.8 × 2.5–5.4 µm. Conidia originate directly from the conidiogenous cells in the long conidiophores and from the separating cells in the short ones, aggregated, dry, straight, teardrop-shaped, truncate at distal end, narrow-tipped, terminal, hyaline, smooth, diaphragm, without a hyaline transverse band, 13.6–22.2 × 3.9–9.8 µm. Sexual morph: Inconclusive.

##### Culture characteristics.

On PDA medium, after seven days of dark incubation in a 25 °C incubator, colonies reached 68 mm in diameter with a growth rate of 9.2–10.2 mm/day. Colonies on PDA raised, concentric, white, flatter, velutinous edge, with mycelium in the middle portion aggregated into a circle; reverse pale yellow, velutinous edge.

##### Additional material studied.

China • Hainan Province, Ledong County, Jianfengling National Forest Park, 18°44'25"N, 108°51'32"E, on diseased leaves of *Myristicafragrans*, 14 October 2023, W.W. Liu, HSAUP 638602, living culture SAUCC638602.

##### Notes.

Based on the phylogenetic tree constructed using ITS and LSU sequences, *Beltraniellamyristicae* emerged as the closest to *B.brevis* DS 2-23 with 94% MLBS and 1.00 BPP support values (Fig. 1). However, a significant discrepancy was observed in the ITS sequence, with a disparity of 7/548 bp between *B.myristicae* and *B.brevis*. Morphologically, *B.myristicae* differed from *B.brevis* by having shorter setae (*B.myristicae*: 80.5–99.8 × 2.7–5.5 vs. *B.brevis*: 89–251 × 4.5–10.5 µm), shorter separating cells (*B.myristicae*: 7.0–12.8 × 2.5–5.4 vs. *B.brevis*: 11–18 × 3.4–4.1 µm), and shorter conidia (*B.myristicae*: 13.6–22.2 × 3.9–9.8 vs. *B.brevis*: 20–26.5 × 4.5–7.2 µm); differences in conidia (*B.brevis*: diamond-shaped, with a hyaline supraequatorial transverse band; *B.myristicae*: teardrop-shaped, without a hyaline transverse band). Consequently, *B.myristicae* was classified as a new species within the genus *Beltraniella*, based on a combination of phylogenetic analysis and morphological comparisons

#### ﻿ Beltraniella xinglongensis

Taxon classificationFungiAmphisphaerialesBeltraniaceae

W.W. Liu, C.Z. Yin, Z.X. Zhang & X.G. Zhang sp. nov.

BE4FCD14-D014-5045-BD2F-AD2C096FFBAC

MB856046

Fig. 5

##### Holotype.

China • Hainan Province, Wanning City, Xinglong tropical botanical garden, 18°43'59"N, 110°11'55"E, on decaying leaves, 24 April 2024, W.W. Liu, holotype HMAS 353196, ex-type living culture SAUCC737701.

**Figure 5.**
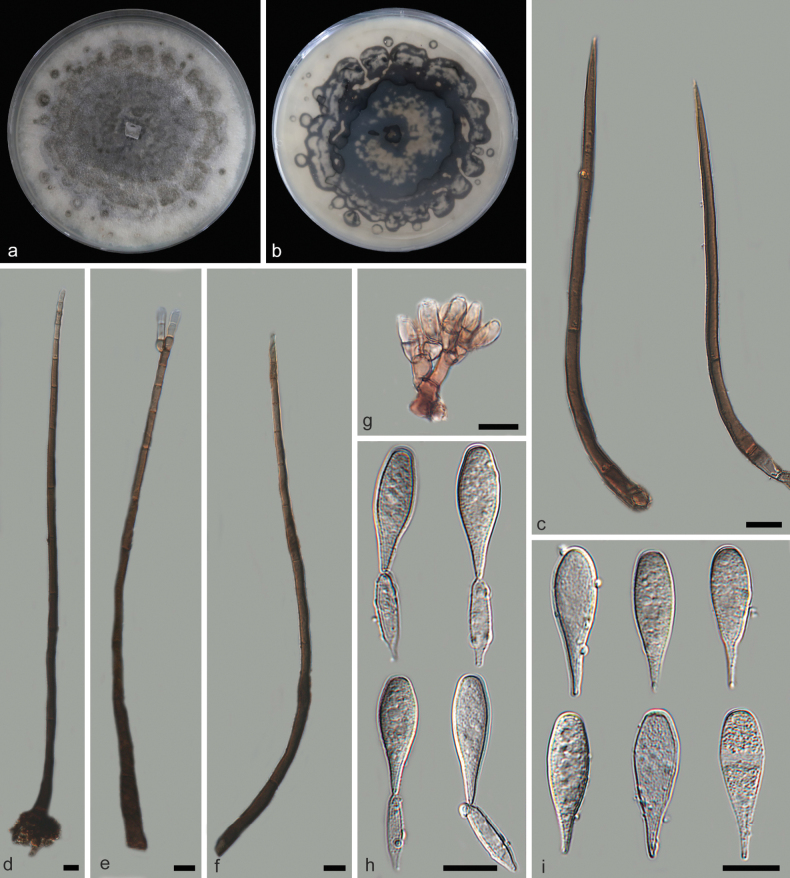
*Beltraniellaxinglongensis* (holotype: HMAS 353196) **a, b** colony front and back after 7 days culture on PDA **c** setae **d–f** long conidiophores **g** short conidiophores **h** separating cells and conidia **i** conidia. Scale bars: 10 μm (**d–i**).

##### Etymology.

The epithet “*xinglongensis*” refers to the name of the location, Xinglong tropical botanical garden where the holotype was collected.

##### Description.

Parasitic on decaying leaves. Asexual morph: Setae dark-brown, unbranched, tapering to a pointed apex, upright, single or in small groups, septate, straight or gently flexuous, emerging from radially lobed basal cells, 75.9–195.9 × 2.5–6.1 μm. Conidiophores septate, occasionally reduced to conidiogenous cells, smooth, swollen at the base, subhyaline to pale brown, present two distinct forms: long and short. Long conidiophores emerging from lobed basal cells, upright, straight or gently curved, simple or branched at apical regions, 13–15 septate, verrucose, swollen at the base, olivaceous to dark-brown, arising from basal cells of setae or from separate, 232.5–298.6 × 2.4–4.9 μm. Short conidiophores hyaline, septate, smooth, 21.2–47.8 × 3.2–6.4 μm. Conidiogenous cells ovoid, polyblastic, cylindrical, hyaline to subhyaline, integrated, denticulate, terminal, smooth, 6.5–9.7 × 2.8–5.4 μm. Separating cells fusiform, ellipsoid to subglobose, smooth, 13.6–17.6 × 2.3–5.4 μm. Conidia originate directly from the conidiogenous cells in the long conidiophores and from the separating cells in the short ones, teardrop-shaped, narrow-tipped, aggregated, terminal, simple, dry, straight, hyaline, smooth and integrated, without a hyaline transverse band, 21.9–28.7 × 5.0–9.5 μm. Sexual morph: Inconclusive.

##### Culture characteristics.

On PDA medium, after seven days of dark incubation in a 25 °C incubator, colonies reached a diameter of 90 mm with a growth rate of 12.5–13.1 mm/day. Colonies on PDA raised, cottony, white, flatter, with gray exudates, sparse aerial mycelia, undulate margin; reverse white, with an undulate margin, abundant gray exudates.

##### Additional material studied.

China • Hainan Province, Wanning City, Xinglong tropical botanical garden, 18°43'59"N, 110°11'55"E, on decaying leaves, 24 April 2024, W.W. Liu, HSAUP 737701, living culture SAUCC737701.

##### Notes.

Based on the phylogenetic tree constructed with ITS and LSU sequence, *Beltranielladujiangyanensis* was the closest relative to *B.xinglongensis*, with a gap of 16/502 bp between their comparative ITS sequences. Morphologically, *B.xinglongensis* differed from *B.dujiangyanensis* in having longer long conidiophores (*B.xinglongensis*: 232.5–298.6 × 2.4–4.9 μm vs. *B.dujiangyanensis*: 113.1–259.9 × 3.1–5.8 μm) and more septa (*B.xinglongensis*: 13–15 septa vs. *B.dujiangyanensis*: 9–10 septa), longer in short conidiophores (*B.xinglongensis*: 21.2–47.8 × 3.2–6.4 μm vs. *B.dujiangyanensis*: 13.1–31.9 × 3.2–5.7 μm), longer separating cells (*B.xinglongensis*: 13.6–17.6 × 2.3–5.4 μm vs. *B.dujiangyanensis*: 9.7–12.3 × 3.1–5.3 μm) and longer conidia (*B.xinglongensis*: 21.9–28.7 × 5.0–9.5 μm vs. *B.dujiangyanensis*: 16.5–21.1 μm). Consequently, *B.xinglongensis* was identified as a new *Beltraniella* species through phylogenetic analysis and morphological comparison

## ﻿Discussion

With the increasing number of reported species within this genus, the classification of *Beltraniella* has encountered significant challenges. The observed striking similarity in spore structures, characterized by turbinate or biconic shapes, often with caudate appendages, as documented in early studies, accounts for this phenomenon (Hudson and Ellis 1972). Consequently, elucidating interspecific relationships within the genus using traditional morphological identification methods has been exceedingly challenging. We employed ITS and LSU to procure fungal DNA sequences, followed by the evaluation of phylogenetic relationships using maximum likelihood (ML) and Bayesian inference (BI) methods. To achieve a comprehensive and accurate classification, we complemented our analysis with morphological assessments (Miller et al. 2010; Stamatakis 2006; Stamatakis et al. 2008). In this investigation, four new species of fungi were identified and reported, namely *Beltranielladujiangyanensis*, *B.jianfengensis*, *B.myristicae*, and *B.xinglongensis*. Based on DNA sequence analysis, these four new species were initially identified as *Beltraniella*. Subsequent phylogenetic analyses with other *Beltraniella* species confirmed their phylogenetic placement with high confidence (Ronquist and Huelsenbeck 2003; Crous et al. 2017; Liu et al. 2019). Subsequently, morphological assessments were conducted to discern the similarities and distinctions among *B.dujiangyanensis*, *B.jianfengensis*, *B.myristicae*, *B.xinglongensis*, and additional *Beltraniella* species within the same clade. The four species, *B.dujiangyanensis*, *B.jianfengensis*, *B.myristicae*, and *B.xinglongensis* were validated as new species within the genus *Beltraniella* based on conventional morphological analysis and molecular phylogenetic analysis. The *Beltraniella* taxon is minute, and a previous study employed phylogenetic analysis integrating ITS and LSU sequences with morphological analysis. The understanding of interspecific relationships has been enriched by the addition of supplementary morphological descriptions. Index Fungorum (https://indexfungorum.org/Names/Names.asp, accessed on 14 February 2025) listed 33 species, of which 25 were recorded in the National Center for Biotechnology Information (NCBI) (https://www.ncbi.nlm.nih.gov/, accessed on 14 February 2025). To ensure data reliability, all pertinent sequences of these 25 species were incorporated.

*Beltraniella* demonstrates a global distribution, supported by the GlobalFungi database (https://globalfungi.com/, accessed on 14 February 2025) comprising 1098 samples and 1626 sequences. Specifically, *Beltraniella* was detected in Asia (57.92%), South America (19.31%), North America (9.11%), Africa (7.1%), Australia (4.28%), Europe (1.73%), Pacific Ocean (0.27%), Antarctica (0.18%), and Indian Ocean (0.09%). The samples used in this study originated from Sichuan and Hainan Provinces, which are characterized by the Central Subtropical Monsoon Humid Climate and the Tropical Rainforest Climate, respectively. These regions are characterized by abundant precipitation, a humid climate, diverse vegetation, and a rich assortment of fungi, including *Beltraniella*. In addition, *Beltraniella* is recognized as an invasive fungus affecting a broad spectrum of plants and has been observed parasitizing diverse plant leaves. For instance, two *Beltraniella* species, *B.botryospora* and *B.portoricensis*, were detected on the deciduous leaves of representative plants from the Atlantic Forest (*Ingathibaudiana*, *Myrciasplendens*, and *Peraglabrata*) (dos Santos et al. 2014). Furthermore, the presence of *B.endiandrae* was verified on the fallen leaves of a Lauraceae plant in Nightcap National Park, New South Wales, Australia. (Crous et al. 2014). Our analysis of *Beltraniella*’s sequence and morphological characteristics revealed its parasitism on diseased leaves of *Myristicafragrans* and decaying leaves. Specifically, *B.dujiangyanensis*, *B.jianfengensis* and *B.xinglongensis* were identified as parasites on decaying leaves, whereas *B.myristicae* was observed to parasitize diseased leaves of *Myristicafragrans*. These findings indicate the potential existence of additional host species of *Beltraniella* among these two hosts (Hyde et al. 2007). Therefore, we expect to discover additional species of *Beltraniella* fungi through the collection of diseased leaves of *Myristicafragrans* and decaying leaves in Hainan and Sichuan Provinces. Ultimately, this research enhances our understanding of fungal diversity in Hainan and Sichuan provinces and expands the known species range of *Beltraniella* fungi.

## ﻿Conclusions

In this study, a wide range of new fungal species were isolated from a large collection of diseased and decaying leaves gathered from Sichuan and Hainan provinces, China. Through rigorous phylogenetic analysis and examination of morphological characteristics, we successfully identified four new species within the genus *Beltraniella*. The pathogenicity and host associations of these newly reported *Beltraniella* fungi are relatively underexplored, necessitating further research. Building on the insights from this study, we anticipate that a more targeted collection of diseased and decaying leaves will expedite the isolation and characterization of further potential *Beltraniella* fungi.

## Supplementary Material

XML Treatment for Beltraniella dujiangyanensis

XML Treatment for Beltraniella jianfengensis

XML Treatment for Beltraniella myristicae

XML Treatment for Beltraniella xinglongensis

## ﻿Additional information

### Author ORCIDs

Wen-Wen Liu https://orcid.org/0009-0009-3477-194X

Chang-Zhun Yin https://orcid.org/0009-0000-0034-2199

Zhao-Xue Zhang https://orcid.org/0000-0002-4824-9716

Xing-Sheng Wang https://orcid.org/0009-0005-1127-7368

Shi Wang https://orcid.org/0000-0002-7376-7638

### Data availability

All of the data that support the findings of this study are available in the main text or Supplementary Information.

## Supplementary materials

Supplementary material 1 DNA sequences Data type fas https://binary.pensoft.net/file/1304098This dataset is made available under the Open Database License (http://opendatacommons.org/licenses/odbl/1.0/). The Open Database License (ODbL) is a license agreement intended to allow users to freely share, modify, and use this Dataset while maintaining this same freedom for others, provided that the original source and author(s) are credited.Wen-Wen Liu, Chang-Zhun Yin, Zhao-Xue Zhang, Xing-Sheng Wang, Zhe Meng, Xiu-Guo Zhang, Shi Wang
